# Effects of broccoli sprout supplements enriched in glucoraphanin on liver functions in healthy middle-aged adults with high-normal serum hepatic biomarkers: A randomized controlled trial

**DOI:** 10.3389/fnut.2022.1077271

**Published:** 2022-12-22

**Authors:** Shohei Satomi, Shingo Takahashi, Kazutaka Yoshida, Sunao Shimizu, Takuro Inoue, Tsuyoshi Takara, Hiroyuki Suganuma

**Affiliations:** ^1^Innovation Division, Department of Diet and Wellbeing Research, KAGOME Co., Ltd., Nasushiobara, Japan; ^2^Medical Corporation Seishinkai, Takara Clinic, Tokyo, Japan

**Keywords:** liver function, alanine aminotransferase (ALT), γ-glutamyl transpeptidase (γ-GTP), glutathione (GSH), sulforaphane (SFN), glucoraphanin, randomized controlled trial (RCT), healthy adults

## Abstract

Sulforaphane (SFN), an isothiocyanate derived from glucoraphanin, has antioxidant, and anti-inflammatory effects that may be beneficial for improving liver function. However, few studies regarding the effects of glucoraphanin on the biological markers related to liver function, such as alanine aminotransferase (ALT), aspartate aminotransferase (AST), and gamma-glutamyltransferase (γ-GTP) in healthy individuals have been reported. This randomized, double-blind, placebo-controlled parallel- group trial was conducted from April 22 to December 25, 2021 and compared the effects of broccoli sprout supplements enriched in glucoraphanin (glucoraphanin supplements) (*n* = 35) with those of placebo supplements (*n* = 35). This trial was registered with the University Hospital Medical Information Network Clinical Trial Registry (UMIN-CTR; ID number UMIN000044005) https://center6.umin.ac.jp/cgi-open-bin/ctr_e/ctr_view. cgi?recptno=R000050252. Glucoraphanin significantly improved serum ALT levels at 24 weeks compared to placebo supplements. However, no significant difference in serum glutathione levels, one of the major antioxidants synthesized in the liver, was observed between the two groups. In conclusion, daily intake of the glucoraphanin supplements is beneficial for maintaining liver health in healthy, middle-aged adults with high-normal serum hepatic biomarkers, although further studies focusing on other antioxidant markers are needed to understand how glucoraphanin improves liver function.

## 1 Introduction

The liver plays an important role in glucose metabolism and glycogen synthesis (1). It also breaks toxins down into low-risk substances, produces, and secretes bile, stores glycogen, and metabolizes drugs (1, 2). Liver damage due to liver diseases (3, 4), drug use (5), excessive alcohol consumption (6), and/or high-energy foods (7) can lead to liver dysfunction that may result in serious diseases, such as liver fibrosis and liver cancers. In addition, liver disease often progresses gradually and may not be diagnosed until a late stage. Therefore, routine liver health examinations are necessary *via* the evaluation of several enzymatic biomarkers, including alanine aminotransferase (ALT), aspartate aminotransferase (AST), and gamma-glutamyltransferase (γ-GTP) (5, 8). Appropriate ranges of serum ALT, AST, and γ-GTP levels are necessary to maintain liver health.

A recent review suggested that glucosinolates and their isothiocyanate metabolites found in cruciferous vegetables are important components in the prevention and treatment of multiple chronic diseases (9). Cruciferous vegetables include arugula (rocket), bok choy, broccoli, Brussels sprouts, cabbage, cauliflower, collard greens, daikon, horseradish, kale, kohlrabi, radish, turnips, wasabi, and watercress and are commonly consumed globally (10). Epidemiological studies showed that higher intakes of these vegetables are associated with a reduced risk of cardiometabolic diseases (11, 12), musculoskeletal conditions (13), and cancer (14). This may be due to the presence of glucosinolates found in cruciferous vegetables, although these vegetables have other nutrients. Glucosinolates (and their isothiocyanates) found in commonly consumed cruciferous vegetables include glucoraphanin (sulforaphane; SFN), sinigrin (allyl isothiocyanate), glucobrassicin, glucoraphasatin, and glucoiberin (15). Among these glucosinolates and isothiocyanates, a large body of work have focused on glucoraphanin and sulforaphane, and the number of studies involving the effects of these on human are increasing (9).

Glucoraphanin is digested by the myrosinase enzyme into SFN in the intestine. SFN has protective effects on the liver, which may be due to its antioxidative and anti-inflammatory functions (16). SFN also induces a series of biological defense genes, including phase II detoxification/antioxidant enzymes and antioxidant molecules such as glutathione (GSH, L-γ-glutamyl-L-cysteinyl-glycine), and suppresses inflammation *via* the inhibition of nuclear factor kappa B (NF-κB) and the activation of the transcription factor nuclear factor-erythroid 2-related factor 2 (Nrf2) (17, 18). GSH plays a crucial role in the antioxidant defense system, demonstrating unique antioxidant properties that improve liver health as 90% of circulating GSH is synthesized in the liver (19). GSH is transported to other organs *via* the circulation (20); therefore, its concentration can be monitored. Several *in vivo* experiments have reported that the administration of SFN or glucoraphanin has a protective effect on various liver diseases that induced by hepatotoxic substances (21), alcohol (22), and high-energy diets (23) *via* Nrf2-mediated mechanisms. A randomized controlled trial (RCT) reported that glucoraphanin intake significantly improves serum ALT and γ-GTP levels in male participants with dyslipidemia and hepatic dysfunction (24). In another previous study (25), the *post hoc* subgroup analyses focusing on healthy participants aged 45–64 years suggested that intake of 54.9 μmol/d (24 mg/d) of glucoraphanin for 24 weeks possibly improve serum ALT levels.

However, whether glucoraphanin intake in healthy adults improves and maintains liver function is not fully understood as these previous studies have several limitations, including focusing on patients with liver diseases (24), *post hoc* subgroup analyses involving decreased detection power, increased alpha error, and loss of accurate randomization, and a lack of information related to the mechanisms by which glucoraphanin functions in the liver (25). To eliminate these limitations, an RCT is needed to focus on the effects of glucoraphanin on antioxidant activity in healthy participants. This RCT investigates the effects of glucoraphanin intake on serum hepatic biomarkers in healthy, middle-aged adults with high-normal serum hepatic biomarkers. This is the first study to investigate the mechanism by which glucoraphanin affects liver function in this participant population.

## 2 Materials and methods

### 2.1 Trial design

A randomized, double-blind, placebo-controlled, parallel-group trial was conducted between April 22-December 25, 2021. This trial was approved by the Research Ethics Review Board of the Medical Corporation Seishinkai, Takara Clinic, and KAGOME CO., LTD. (approval numbers 2104-04535-0033-31-TC and 2021-R01, respectively), and registered with the University Hospital Medical Information Network Clinical Trial Registry (UMIN-CTR; ID number UMIN000044005). This trial involving human subjects and the Declaration of Helsinki was conducted in accordance with the ethical guidelines for medical and biological research.

**FIGURE 1:**
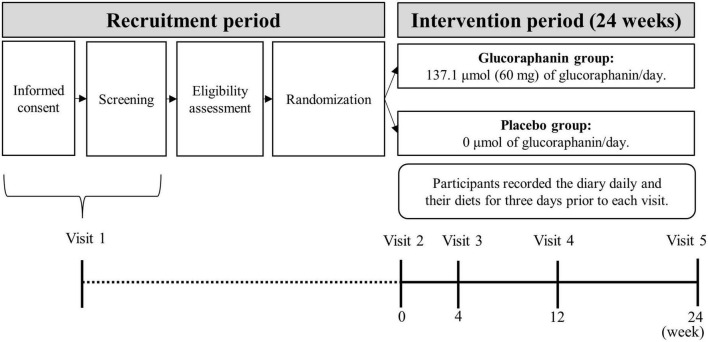
Outline of the trial design^1^. ^1^Visit dates: Visit 1; between April 22nd and July 3rd, Visit 2; between May 17th and July 10th; Visit 3: between June 14th and August 7th; Visit 4: between August 9th and October 2nd; Visit 5: between November 1st and December 25th. Each visit was conducted in 2021.

Figure 1 shows an outline of the trial design. We entrusted ORTHOMEDICO, Inc. (Tokyo, Japan), a Contract Research Organization (CRO), with conducting the trial. Trial participants were recruited *via* a recruiting website^1^ operated by ORTHOMEDICO Inc. Written informed consent was obtained from all participants before the screening survey between April 22nd and July 3rd (visit 1). After the participants were screened for trial eligibility, they were randomly allocated to the glucoraphanin group or placebo group at an allocation ratio of 1:1 based on sex (male/female) and ALT level (the primary outcome) measured at screening survey (described in section “2.2.5 Randomization and allocation concealment”). Immediately before the intervention started, each participant visited the Takara Clinic (Tokyo, Japan) or Nerima Medical Association Minami-machi Clinic (Tokyo, Japan) between May 17th and July 10th (week 0; visit 2). After visit 2, each participant consumed six glucoraphanin capsules (22.9 μmol (10 mg) glucoraphanin each; 137.1 μmol (60 mg) total) or six placebo capsules (0 μmol glucoraphanin) daily for 24 weeks. During the intervention, each participant visited the clinic at week 4 (visit 3; 4 weeks after the start of the intervention) between June 14th and August 7th, at week 12 (visit 4; 12 weeks after the start of the intervention) between August 9th and October 2nd, and at week 24 (visit 5; 24 weeks after the start of the intervention) between November 1st and December 25th. The participants recorded a diary daily and their diet for 3 days before each of five visits throughout the study period (described in section “2.4.1 Diary and diet records”). The participants completed a medical questionnaire and underwent physical examinations, blood sampling, and urine sampling at each visit.

### 2.2 Participants

#### 2.2.1 Inclusion, eligibility, and exclusion criteria

All participants were healthy Japanese residents aged between 45 and 65 years. Participants who were deemed eligible to participate in the trial by their principal doctor and had serum ALT levels 31 ≤ ALT ≤ 50 U/L, serum AST levels ≤ 50 U/L, and serum γ-GTP levels ≤ 100 U/L were included in the trial. In Japan, serum ALT>50, AST>50, and γ-GTP> 100 (U/L) are considered unhealthy, and serum ALT ≤ 30, AST ≤ 30, and γ-GTP ≤ 50 (U/L) are used as reference values (26). Therefore, the participants in this trial had ALT levels on the high end of the normal range.

Participants with a history of or who were currently undergoing treatment for serious illnesses (malignant tumors, heart failure, or myocardial infarction) and those with pacemakers or implantable defibrillators, cardiac arrhythmia, hepatic disease, renal disease, cerebrovascular disease, rheumatism, diabetes mellitus, dyslipidemia, hypertension, hepatitis B, hepatitis C, or other chronic diseases were excluded from the trial. In addition, participants who reported routinely drinking 60 g or more pure alcohol daily, routinely eating foods for specified health use, foods with functional claims, or other health foods, or routinely taking pharmaceuticals and supplements were also excluded from the study. Participants with allergies to medications or supplements including broccoli, shrimp, crab, eggs, wheat, buckwheat, peanuts, or milk ingredients and those who were pregnant and/or lactating, including those who were planning a pregnancy during the trial, were excluded from the trial. Finally, those participating in other clinical trials for 28 days before the date of obtaining consent for this trial (including those who planned to participate to other clinical trials during the trial) and those deemed ineligible to participate in the study by their principal doctor were excluded from this trial.

#### 2.2.2 Dropout and stopping guidelines

Participants were considered trial dropouts when they voluntary withdrew for personal reasons, did not adhere to the procedure required by the study investigators and/or persons responsible for the trial operation, deviated from the lifestyle described in the study protocol, or were deemed ineligible by their principal doctor.

Participants were considered as stopped when a serious adverse event occurred that required them to stop participating in the trial, the participant’s principal doctor detected objective symptoms that would impede the participant’s ability to continue with the trial, or it was considered difficult for the participant to continue the trial by their principal doctor due to any reason.

#### 2.2.3 Participant protocol

The participants were required to consume the prescribed amounts of experimental supplements throughout the trial at a rate of ≥ 90% and avoiding excessive food and alcohol intake. The participants were also required to maintain their lifestyle throughout the trial (from the day of signed informed consent to the study completion; 24 weeks after the study initiation) and avoid drinking alcohol and excessive exercise from the day before each visit until the end of the examination at each visit. The consumption of foods with specific health uses or functional claims and other health foods (such as those used to improve and/or maintain health) was prohibited. The participants were not permitted to eat (including experimental supplements) or drink (except for water) 6 h prior to blood sampling. In addition, the participants were instructed to contact the CRO (ORTHOMEDICO, Inc.) without delay if an adverse event occurred.

#### 2.2.4 Sample size calculation

To determine the required sample size, a simulation using information from a previous clinical trial was conducted (25). The stratified analysis in the previous trial focusing on participants aged 45–65 years with ALT levels 31 ≤ ALT ≤ 50 (U/L) showed that the mean difference and the integrated standard deviation between the glucoraphanin group and the placebo group were 9.0 and 9.6 U/L, respectively. For a statistical significance level (α) of 5% and a statistical power (1 - β) of 95%, 60 participants (30 in each group) are required. Furthermore, considering the dropout rate and non-adherence to the protocol and/or directions, the initial number of study participants was set at 80 (40 in each group).

#### 2.2.5 Randomization and allocation concealment

An allocation manager, who was not directly involved in the study, equally but randomly assigned the participants to either the glucoraphanin group (*n* = 38) or placebo group (*n* = 40) using stratified block random allocation based on sex (male/female) and ALT level (the primary outcome) using SPSS Statistics (version 23; IBM Japan, Ltd., Tokyo, Japan). Allocation was performed according to a computer-generated randomization list. The allocation manager created an allocation table that was concealed until the completion of the data analysis. No individual who was related to the trial, except the allocation manager, was aware of the group assignments.

### 2.3 Experimental supplements

Each participant consumed either six glucoraphanin capsules or six placebo capsules per day with approximately 100 mL of water after breakfast throughout the trial intervention period. The glucoraphanin capsules contained broccoli sprout extracts, cornstarch, dextrin, onion powder, ginger powder, parsley powder, hydroxypropyl methylcellulose, crystalline cellulose, and calcium stearate. Six capsules of glucoraphanin supplements provided 6 kcal, 0.14 g protein, 0.004 g fat, and 137.1 μmol glucoraphanin. The placebo capsules contained the same ingredients as the glucoraphanin capsule, except for broccoli sprout extracts. Therefore, they contained 0 μmol glucoraphanin. Each participant received total of 12 aluminum pouches containing 93 capsules/pouch. The supplements were indistinguishable in appearance, shape, color, odor, and taste. Taking participant’s burden into consideration, we employed 137.1 μmol/d (6 capsules/d) of glucoraphanin as the maximum of oral doses.

### 2.4 Measurements

#### 2.4.1 Diary and diets records

The participants recorded their daily intake of experimental supplements (yes/no), alcohol consumption (yes/no), amount of alcohol consumption, and menstruation (yes/no; female only) in a daily diary during the intervention period. The examples of type of alcohol and its contents were provided to the participants as follows: beer (500 mL, 5%) provided 20 g of pure alcohol, Japanese sake (180 mL, 15%) provided 22 g of pure alcohol, whiskey brandy (double 60 mL, 43%) provided 20 g of pure alcohol, Japanese Shochu (180 mL per cup, 25%) provided 36 g of pure alcohol, and wine (120 mL per cup, 12%) provided 12 g of pure alcohol. One aluminum pouch containing surplus capsules was collected by mail once every 2 weeks, and the compliance with dose of the supplements was confirmed by pill counts. The diaries were submitted weekly by mail except during visit 5, at which they submitted their diary from the previous week in person or completed it at the visit. The participants recorded their diets for 3 days prior to each visit *via* the Calorie and Nutrition Diary (CAND^®^) (27), developed by ORTHOMEDICO Inc. The participants submitted their diet records at the time of each visit or completed it during the visit.

#### 2.4.2 Medical questionnaire

The participants’ physical conditions were assessed *via* a medical questionnaire during each visit.

#### 2.4.3 Physical examination

The participants’ height was measured using a height meter (Tsutsumi Corporation, Tokyo, Japan), and their weight and body fat percentage were measured using a body composition meter [X-SCAN PLUS (Sowa Medical Corporation, Fukuoka, Japan) or MC-780A-N (TANITA CORPORATION, Tokyo, Japan)]. The body mass index (BMI) was calculated by dividing the body weight (kg) by the height squared (m^2^). The systolic blood pressure, diastolic blood pressure, and pulse rate were measured using a sphygmomanometer (HEM-6022; OMRON HEALTHCARE Co., Ltd., Kyoto, Japan). Physical examinations were performed at visit 1 and/or visit 2.

#### 2.4.4 Urinary examination

Approximately 10 mL of urine was collected from each participant during each visit. The urine protein, glucose, urobilinogen, bilirubin, ketone bodies, pH, and occult blood were measured. Urinary examinations were outsourced to LSI Medience Corporation (Tokyo, Japan).

#### 2.4.5 Hematological examination

Approximately 16 mL of venous blood was collected from each participant during each visit. The leukocyte count, erythrocyte count, hemoglobin, hematocrit value, platelet count, mean corpuscular volume, mean corpuscular hemoglobin, mean corpuscular hemoglobin concentration, percentage, and number of neutrophils, lymphocytes, monocytes, eosinophils, and basophils, AST, ALT, γ-GTP, alkaline phosphatase, lactate dehydrogenase, leucine aminopeptidase, total bilirubin, direct bilirubin, indirect bilirubin, cholinesterase, total protein, urea nitrogen, creatinine, uric acid, creatine kinase, sodium, potassium, chloride, calcium, inorganic phosphorus, serum iron, serum amylase, total cholesterol, high density lipoprotein cholesterol, low density lipoprotein cholesterol, triglyceride, glucose, HbA1c, GSH, and non-specific immunoglobulin E (IgE) were analyzed. IgE levels were measured during visit 1, while the other parameters were measured at each visit. Hematological examinations, except for GSH, were outsourced to the LSI Medience Corporation. The measurement of GSH was outsourced to Nikken SEIL Co., Ltd. (Shizuoka, Japan).

### 2.5 Endpoints

#### 2.5.1 Primary endpoint

The primary endpoint was serum ALT at 24 weeks (visit 5).

#### 2.5.2 Secondary endpoints

The secondary endpoints were the serum ALT at 4 weeks (visit 3) and 12 weeks (visit 4), the serum AST and γ-GTP at 4 weeks (visit 3), 12 weeks (visit 4), and 24 weeks (visit 5), and the serum GSH at 4 weeks (visit 3), 12 weeks (visit 4), and 24 weeks (visit 5).

### 2.6 Safety analysis

The incidences of side effects and adverse events during the intervention period were evaluated. The measurement items, except for the primary and secondary endpoints, in the hematological examination were evaluated.

### 2.7 Statistical analyses

All statistical analyses were performed using SPSS version 23.0 software (IBM Japan Ltd., Tokyo, Japan). The values of measurements at visit 2 were defined as baseline values. The differences in ALT, AST, γ-GTP, and GSH levels between the two groups were analyzed using Welch’s *t*-test or a linear mixed model fitted to the following covariates: fixed effects for categorical variables including group, time point, and group-time point interactions; and fixed effects for continuous variables including baseline values and baseline values-time point interactions. Logarithmic values were used for statistical analyses when the data was not distributed normally. For safety analysis, the differences in the incidence of side effects and adverse events between the two groups were analyzed using the chi-square test. The measurement items, except for the primary and the secondary endpoints, in the hematological examination were analyzed using Welch’s *t*-test. In all analyses, *p*-values < 0.05 were considered statistically significant.

## 3 Results

### 3.1 Flow diagram and participant characteristics

**FIGURE 2:**
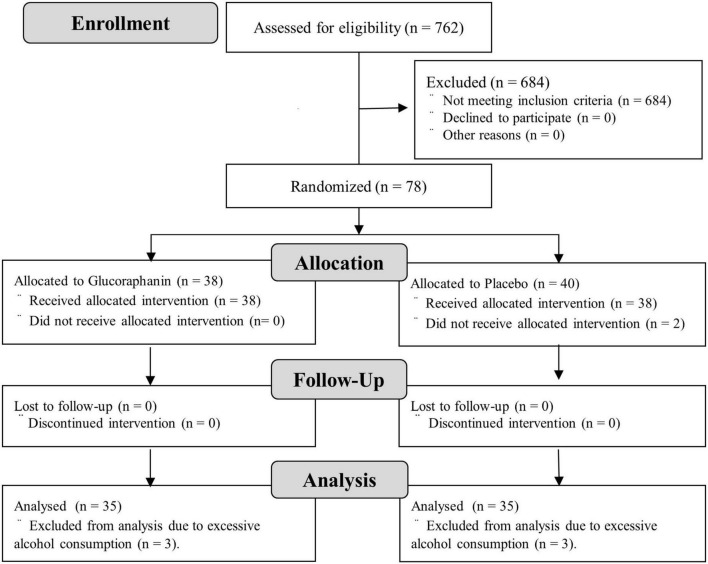
Flow diagram of trial participants.

Figure 2 shows a flow diagram of the trial participants. A total of 762 potential participants were screened for trial eligibility, and 684 did not meet the inclusion criteria. Among 684 potential participants excluded in the trial eligibility, 678 did not meet the liver function biomarkers. Therefore, 78 participants were enrolled in this trial and randomly allocated to the glucoraphanin (*n* = 38) or the placebo (*n* = 40) groups. Two participants in the placebo group did not consume the capsules. Therefore, the safety analyses included 76 participants. In addition, three participants in each group reported excessive alcohol intake. Therefore, the efficacy analyses included 70 participants. The adherence rates to the experimental supplements were 99.2% for the glucoraphanin group (*n* = 35) and 99.3% for the placebo group (*n* = 35). The participant’s characteristics at visit 1 were not significantly different between the two groups (Table 1).

**TABLE 1:** Participant characteristics.

Variable (unit)	Glucoraphanin (*n* = 35)	Placebo (*n* = 35)	*P*-value
Sex (male/female)	26/9 (74.3%)	26/9 (74.3%)	1.0
Age (years)	52.7 (5.3)	52.5 (4.8)	0.89
Height (cm)	168.9 (8.2)	165.6 (6.2)	0.063
Weight (kg)	72.3 (15.7)	70.1 (12.8)	0.52
BMI (kg/m^2^)	25.1 (4.2)	25.4 (3.7)	0.75
Body fat (%)	25.8 (8.5)	26.2 (5.6)	0.84
Systolic blood pressure (mmHg)	127.9 (17.5)	126.7 (15.5)	0.75
Diastolic blood pressure diastolic (mmHg)	85.6 (12.7)	84.9 (11.4)	0.79
Heart rate (bpm)	76.3 (11.7)	74.0 (12.1)	0.42
ALT (U/L)	38.0 (33.5–44.0)	39.0 (35.0–46.5)	0.37
AST (U/L)	28.0 (24.0–33.0)	29.0 (24.5–33.5)	0.44
γ-GTP (U/L)	34.0 (27.5–52.5)	44.0 (31.0–68.5)	0.21

Data are expressed as numbers (percentage) or mean (standard deviation).

ALT, AST, and γ-GTP values are expressed as median (interquartile range).

The chi-square test was used to compare sex, and *t*-tests were used to compare other variables.

ALT, alanine aminotransferase; AST, aspartate aminotransferase; γ-GTP, gamma-glutamyl transferase; BMI, body mass index.

### 3.2 ALT, AST, γ-GTP, and GSH

Serum ALT, AST, γ-GTP, and GSH levels at zero, four, 12, and 24 weeks (visits 2–5) are summarized in Table 2. These biomarkers were not distributed normally (data not shown). The serum ALT levels were significantly lower in the glucoraphanin group than in the placebo group at 24 weeks (*p* = 0.041). Although not significant, the serum γ-GTP levels tended to be lower in the glucoraphanin group than in the placebo group at 24 weeks (*p* = 0.088). No other significant differences were noted between the two groups. Individual plots in ALT levels were shown in Supplementary Figure 1. This also confirmed that ALT levels in glucoraphanin group decreased at 24 weeks (visit 5), but not in placebo group.

**TABLE 2:** Serum ALT, AST, γ-GTP and GSH levels at each visit.

Variable (unit)	Visit	Glucoraphanin group (*n* = 35)	Placebo group (*n* = 35)	*P*-value
ALT (U/L)	Visit 2^†^	36.0 (30.0–40.0)	35.0 (27.5–42.0)	0.842
	Visit 3^‡^	31.0 (27.0–36.5)	33.0 (28.5–44.0)	0.145
	Visit 4^‡^	32.0 (28.0–40.0)	34.0 (25.0–41.5)	0.947
	Visit 5^‡,§^	29.0 (22.0–36.5)	30.0 (25.5–41.5)	0.041*
AST (U/L)	Visit 2^†^	28.0 (21.0–30.5)	28.0 (22.0–33.0)	0.696
	Visit 3^‡^	25.0 (23.0–29.5)	26.0 (22.5–33.0)	0.248
	Visit 4^‡^	25.0 (21.5–29.5)	27.0 (22.0–31.5)	0.212
	Visit 5^‡^	26.0 (21.0–31.0)	25.0 (22.0–34.5)	0.348
γ-GTP (U/L)	Visit 2^†^	30.0 (26.5–47.0)	41.0 (27.0–68.5)	0.198
	Visit 3^‡^	31.0 (26.0–50.0)	43.0 (29.0–62.5)	0.774
	Visit 4^‡^	34.0 (25.0–54.0)	46.0 (28.5–61.0)	0.786
	Visit 5^‡^	33.0 (23.5–43.0)	45.0 (26.0–70.0)	0.088^#^
GSH (μM)	Visit 2^†^	3.90 (3.60–4.15)	4.20 (3.80–4.40)	0.179
	Visit 3^‡^	4.30 (3.50–4.65)	4.20 (3.10–4.90)	0.506
	Visit 4^‡^	4.80 (4.60–4.90)	4.80 (4.70–5.00)	0.627
	Visit 5^‡^	5.10 (4.90–5.20)	5.00 (5.00–5.10)	0.953

Data are presented as median (interquartile range).

^†^The differences between the groups were determined using Welch’s *t*-test.

^‡^The differences between the groups were determined using a linear mixed model fitted to the following covariates: fixed effects for categorical variables including group, time point, and group-time point interactions; and fixed effects for continuous variables including baseline values and baseline values-time point interactions.

^§^Primary endpoint.

*p < 0.05.

^#^*p* < 0.1.

ALT, alanine aminotransferase; AST, aspartate aminotransferase; γ-GTP, gamma-glutamyl transferase GSH, glutathione.

### 3.3 Safety analysis

No side effects or serious adverse events were observed in this study (Table 3). Two participants in the glucoraphanin group experienced adverse events including dry eye, headache, and eczema. Eight participants in the placebo group experienced adverse events, including pruritus, feeling cold, muscle pain, eczema, tinea pedis, backache, dry skin, fever, indigestion, diarrhea, headache, dental treatments, fatigue, dry eye, gastritis, eyestrain, and allergic rhinitis. The principal doctor believed that there were no causal relationships between the experimental supplements and these adverse events. Any of the measurements in the hematological examinations was not significantly different between two groups (Supplementary Table 1).

**TABLE 3:** Incidences of adverse effects.

	Glucoraphanin group (*n* = 38)	Placebo group (*n* = 38)	*P*-value
Side effects	0 (0%)	0 (0%)	N.A.
Adverse events	2 (5.3%)	8 (21.1%)	0.086

Data are shown as number (percentage).

*P*-values were determined using the chi-squared test.

N.A., not available.

## 4 Discussion

This trial investigated the effects of glucoraphanin on the serum biomarkers of liver function based on GSH levels in healthy, middle-aged participants with high-normal baseline ALT levels. The intake of 137.1 μmol/d of glucoraphanin over 24 weeks improved the serum ALT levels in healthy Japanese adults without any associated adverse events, although a significant increase in serum GSH levels was not observed.

This RCT included healthy adults and a large sample size, overcoming the limited generalizability and *post hoc* subgroup analyses including small sample sizes of previous studies (24, 25).

In this trial, 137.1 μmol/d of glucoraphanin significantly improved the participants’ serum ALT levels, though the serum AST levels were not affected. These results are consistent with the results of the subgroup analysis in a previous trial in which participants consumed 54.9 μmol/d of glucoraphanin for 24 weeks (25). This suggests that glucoraphanin at the oral doses volume between 22.9 and 137.1 μmol/d similarly might improve ALT levels in healthy middle-aged adults with high-normal serum hepatic biomarkers. ALT is the most commonly used liver damage biomarker worldwide (28) as it has relatively low concentrations in other tissues and is more specific to hepatocellular injury than AST (29). In contrast, AST is non-specific for hepatocellular injury as it is present in skeletal muscle, heart muscle, and kidney tissue (29). Therefore, this study indicates that glucoraphanin can help improve serum ALT levels specific to liver damage.

Furthermore, this trial also found that 137.1 μmol of glucoraphanin tended to improve the participants’ serum γ-GTP levels, which is similar with the results of a previous RCT (24). In the previous RCT, the improved γ-GTP correlated with the reduction of the oxidative stress marker urinary 8-hydroxy-2′-deoxyguanosine in patients with fatty liver. These data indicate that glucoraphanin likely has a positive effect on γ-GTP levels in healthy adults. To confirm the effects of glucoraphanin on γ-GTP, an RCT that strictly controls alcohol consumption should be conducted. Serum γ-GTP levels are reported to increase in response to oxidative stress caused by fatty liver diseases and alcohol consumption (30). The current trial required that participants limited their alcohol consumption to < 60 g of pure alcohol, though this was controlled and reported by the participants. Differences in alcohol intake may have influenced the effects of glucoraphanin on serum γ-GTP in the current trial.

Although glucoraphanin exerted positive effects on liver health in this trial, the mechanisms of glucoraphanin intervention were not clarified as no significant differences in serum GSH levels were observed between the two groups. This may be due to inadequate glucoraphanin administration for GSH induction. A previous *in vivo* study showed that the administration of SFN (at least 2.82 μmol/kg) increases GSH- and glutathione-related enzymes in the liver of liver-aging-induced rats (31). When this amount of SFN is extrapolated to humans, 17.5 μmol/kg SFN are required. However, only 1.90 μmol/kg glucoraphanin (137.1 μmol/72.3 kg average weight in the glucoraphanin group) were administered in this trial. In addition, a previous clinical trial reported that bioavailability, as measured by urinary excretion of SFN and its metabolites (in approximately 12-h collections after dosing), was substantially greater with the SFN (mean = 70%) than with glucoraphanin (mean = 5%) beverages (32). These suggested that much more glucoraphanin may be required to increase serum GSH. While a previous pilot study reported that a 1 week of SFN intake significantly increased GSH in healthy human blood cells (33), a previous review reported that SFN-induced GSH was not always consistent (34). Therefore, the relationship between glucoraphanin or SFN intake and GSH should be investigated further in future studies.

This trial focused on GSH as an important factor protecting against oxidative stress in the liver as it is a unique antioxidant synthesized mainly in liver (19, 35). However, enzymatic antioxidants as well as non-enzymatic antioxidant systems such as GSH are essential for cellular responses to oxidative stress under physiological conditions (36). Antioxidant enzymes, such as catalase, superoxide dismutase, and GSH-peroxidase, are also affected and used as indices to evaluate the level of oxidative stress (36, 37). Regarding the other anti-inflammatory and antioxidants effects, some research previously provided the evidence that there was the relationship between glucoraphanin or sulforaphane and nitric oxide (NO) or malondialdehyde (MDA). A clinical trial showed that high sulforaphane broccoli sprout may attenuate undesirable overproduction of NO in *Helicobacter pylori* infected patients (38). A pilot study investigated the effect of a sulforaphane supplement on muscle soreness and damage induced by eccentric exercise in young adults. Serum MDA was shown to be significantly lower levels 2 days after exercise in the sulforaphane group compared with the control group (39). Future studies should focus on the effects of glucoraphanin intake on these antioxidant enzymes and anti-inflammatory substances.

The results of this trial indicate that 137.1 μmol/d of glucoraphanin for 24 weeks did not lead to adverse events, which is consistent with previous studies in which 54.9 μmol of glucoraphanin were consumed daily for eight (24) or 24 (25) weeks. In this trial, the glucoraphanin dose was more than double that of previous trials. A previous clinical trial reported no adverse effects on the thyroid gland after the daily consumption of a broccoli sprout extract beverage (600 μmol/d of glucoraphanin) for 12 weeks (40). However, this is the first study to report that the intake of high doses (137.1 μmol/d) of glucoraphanin in the form of supplements for a long period (24 weeks) may be useful for liver health without safety concerns.

This study is not without limitations. First, there is a lack of information regarding the mechanisms by which glucoraphanin improves liver function. Second, the complete glucosinolate profile of broccoli sprout extracts was not clarified in this study. This supplement may contain other glucocinolates than glucoraphanin such as glucoiberin, glucoerucin, 4-methylthiobutylblucosinolate and some indole glucosinolates. However, previous reports have shown that glucoraphanin accounts for about 70–90% of glucosinolates in broccoli sprouts (41, 42) or seeds (43) and is thought to be the major glucosinolate in broccoli sprout extracts. Glucoraphanin is converted to SFN *in vivo*, and SFN is known as one of the most potent inducers of Nrf2 among isothiocyanates (44); hence, it is likely that glucoraphanin in broccoli sprout contributed to the Nrf2 activation and protection to liver damage. However, further preclinical and clinical studies might be needed to investigate the effects of other kinds of glucosinolates on liver health. Third, there is a lack of information regarding the effects of the other ingredients in supplements on the ALT levels. Both of supplements contained onion powder, ginger powder and parsley powder. The contents of these powders were totally lower than those of broccoli sprout extracts. It remains unclear whether these powders except for the broccoli sprout extracts up-regulate the Nrf2 pathway, although the difference in ALT levels between the glucoraphanin group and the placebo group was brought by the broccoli sprout extracts since both of supplements contained the same formula of onion powder, ginger powder and parsley powder. Forth, the effects of glucoraphanin intake in younger individuals are unclear, as this trial included participants aged 45–65 years. Therefore, an RCT including a wider age range of participants is needed.

## 5 Conclusion

Daily intake of glucoraphanin supplements safely improves serum ALT levels in healthy, middle-aged adults with high-normal serum hepatic biomarkers. However, further preclinical and clinical studies are needed to confirm the mechanisms by which glucoraphanin improves liver health.

## Data availability statement

The datasets presented in this article are not readily available because de-identified data described in the article, code book, and analytic codes will be made available upon request pending approval of an application for data use and execution of a data use agreement and/or material transfer agreement with KAGOME Co., Ltd. Requests to access the datasets should be directed to SS, Shohei_Satomi@kagome.co.jp.

## Ethics statement

The studies involving human participants were reviewed and approved by the Research Ethics Review Board of the Medical Corporation Seishinkai, Takara Clinic, and KAGOME Co., Ltd. The patients/participants provided their written informed consent to participate in this study.

## Author contributions

SSa, ST, KY, SSh, and TI: conceptualization, methodology, validation, investigation, data curation, and writing original draft. TT: considering adverse events as the principal doctor. HS: project administration and supervision. All authors have read and agreed to the published version of the manuscript.

## Footnotes

1
https://www.go106.jp/
