# Supplementary material for: Effects of broccoli sprout supplements enriched in glucoraphanin on liver functions in healthy middle-aged adults with high-normal serum hepatic biomarkers: A randomized controlled trial
Source: Front Nutr. 2022 Dec 22;9:1077271. doi: 10.3389/fnut.2022.1077271 (PMC9813215; doi:10.3389/fnut.2022.1077271)
Supplement: Supplementary file 1 [file Table_1.DOCX]

Supplementary Material

| **Supplementary table 1** The measurements in the hematological examination at each visit. | | | | |
| --- | --- | --- | --- | --- |
|  | **Visit** | **Glucoraphanin group (n = 38)** | **Placebo group (n = 38)** | ***p*-Value** |
| Leukocyte count (/μL) | Visit 2 | 5950.0 (1660.1) | 5692.1 (1303.9) | 0.454 |
|  | Visit 3 | 6165.8 (2243.2) | 5802.6 (1656.8) | 0.425 |
|  | Visit 4 | 5836.8 (1512.7) | 5723.7 (1682.7) | 0.759 |
|  | Visit 5 | 5926.3 (1564.2) | 5807.9 (1600.6) | 0.745 |
| Erythrocyte count (×10^4^/μL) | Visit 2 | 490.6 (46.1) | 477.9 (44.7) | 0.227 |
|  | Visit 3 | 487.7 (47.4) | 480.9 (46.1) | 0.530 |
|  | Visit 4 | 488.2 (47.1) | 481.0 (46.4) | 0.503 |
|  | Visit 5 | 495.2 (41.3) | 483.5 (45.8) | 0.248 |
| Hemoglobin (g/dL) | Visit 2 | 14.9 (1.2) | 14.7 (1.2) | 0.558 |
|  | Visit 3 | 14.8 (1.3) | 14.9 (1.3) | 0.935 |
|  | Visit 4 | 14.8 (1.2) | 14.8 (1.3) | 0.863 |
|  | Visit 5 | 15.0 (1.2) | 14.9 (1.4) | 0.768 |
| Hematocrit (%) | Visit 2 | 46.2 (3.7) | 45.7 (3.4) | 0.552 |
|  | Visit 3 | 46.0 (3.7) | 46.1 (3.6) | 0.870 |
|  | Visit 4 | 46.2 (3.9) | 45.9 (3.7) | 0.749 |
|  | Visit 5 | 46.6 (3.5) | 46.3 (3.9) | 0.739 |
| Platelet count (×10^4^/μL) | Visit 2 | 25.5 (5.2) | 25.9 (4.4) | 0.733 |
|  | Visit 3 | 25.8 (5.7) | 25.8 (4.5) | 0.970 |
|  | Visit 4 | 25.0 (5.2) | 26.4 (5.0) | 0.218 |
|  | Visit 5 | 25.6 (5.2) | 26.7 (5.0) | 0.362 |
| MCV (fL) | Visit 2 | 94.4 (3.6) | 95.9 (4.2) | 0.085 |
|  | Visit 3 | 94.5 (3.8) | 96.1 (4.1) | 0.083 |
|  | Visit 4 | 94.7 (4.0) | 95.6 (3.7) | 0.314 |
|  | Visit 5 | 94.2 (4.1) | 95.9 (4.0) | 0.063 |
| MCH (pg) | Visit 2 | 30.4 (1.1) | 30.9 (1.5) | 0.133 |
|  | Visit 3 | 30.5 (1.1) | 31.0 (1.4) | 0.113 |
|  | Visit 4 | 30.4 (1.1) | 30.8 (1.4) | 0.231 |
|  | Visit 5 | 30.3 (1.0) | 30.9 (1.6) | 0.071 |
| MCHC (%) | Visit 2 | 32.2 (0.8) | 32.2 (0.8) | 0.882 |
|  | Visit 3 | 32.3 (0.7) | 32.2 (0.7) | 0.751 |
|  | Visit 4 | 32.1 (0.7) | 32.2 (0.8) | 0.742 |
|  | Visit 5 | 32.2 (0.9) | 32.2 (1.0) | 1.000 |
| Neutrophils (%) | Visit 2 | 58.1 (8.0) | 56.3 (8.3) | 0.331 |
|  | Visit 3 | 58.0 (7.6) | 57.0 (8.3) | 0.600 |
|  | Visit 4 | 56.5 (8.1) | 57.2 (8.2) | 0.743 |
|  | Visit 5 | 57.5 (7.0) | 58.6 (8.5) | 0.539 |
| Lymphocytes (%) | Visit 2 | 32.2 (6.9) | 34.0 (8.0) | 0.297 |
|  | Visit 3 | 32.2 (7.1) | 33.4 (7.8) | 0.486 |
|  | Visit 4 | 33.1 (6.7) | 33.0 (8.0) | 0.983 |
|  | Visit 5 | 32.8 (6.5) | 32.3 (8.2) | 0.779 |
| Monocytes (%) | Visit 2 | 5.7 (1.4) | 5.6 (1.1) | 0.758 |
|  | Visit 3 | 5.8 (1.6) | 5.8 (1.4) | 0.938 |
|  | Visit 4 | 5.6 (1.3) | 5.6 (1.2) | 0.978 |
|  | Visit 5 | 5.7 (1.2) | 5.3 (1.1) | 0.217 |
| Data are expressed as mean (SD). The differences between the groups were determined using Welch’s t-test.  Abbreviations: MCV, mean corpuscular volume; MCH, mean corpuscular hemoglobin; MCHC, Mean corpuscular hemoglobin concentration | | | | |
| **Supplementary table 1.** Continued. | | | | |
|  | **Visit** | **Glucoraphanin group (n = 38)** | **Placebo group (n = 38)** | ***p*-Value** |
| Eosinophils (%) | Visit 2 | 3.2 (2.0) | 3.2 (2.1) | 0.867 |
|  | Visit 3 | 3.2 (2.1) | 3.0 (2.0) | 0.564 |
|  | Visit 4 | 4.0 (3.2) | 3.4 (3.1) | 0.416 |
|  | Visit 5 | 3.3 (2.2) | 3.0 (2.1) | 0.567 |
| Basophils (%) | Visit 2 | 0.8 (0.5) | 0.9 (0.4) | 0.759 |
|  | Visit 3 | 0.8 (0.4) | 0.9 (0.4) | 0.522 |
|  | Visit 4 | 0.8 (0.4) | 0.9 (0.4) | 0.815 |
|  | Visit 5 | 0.8 (0.4) | 0.8 (0.4) | 0.885 |
| Neutrophils (/μL) | Visit 2 | 3525.3 (1295.4) | 3226.9 (992.9) | 0.264 |
|  | Visit 3 | 3643.6 (1737.5) | 3364.5 (1287.2) | 0.429 |
|  | Visit 4 | 3356.7 (1161.8) | 3338.9 (1378.9) | 0.952 |
|  | Visit 5 | 3452.1 (1154.0) | 3456.5 (1310.7) | 0.988 |
| Lymphocytes (/μL) | Visit 2 | 1867.0 (534.5) | 1923.6 (613.8) | 0.670 |
|  | Visit 3 | 1928.1 (566.3) | 1896.1 (574.1) | 0.808 |
|  | Visit 4 | 1894.3 (528.2) | 1832.6 (504.6) | 0.604 |
|  | Visit 5 | 1918.0 (560.5) | 1836.5 (549.1) | 0.524 |
| Monocytes (/μL) | Visit 2 | 327.9 (88.4) | 312.8 (71.0) | 0.414 |
|  | Visit 3 | 355.8 (186.4) | 326.1 (94.2) | 0.384 |
|  | Visit 4 | 318.6 (87.5) | 309.6 (75.5) | 0.635 |
|  | Visit 5 | 327.2 (78.7) | 303.4 (77.0) | 0.188 |
| Eosinophils (/μL) | Visit 2 | 181.5 (107.6) | 180.6 (113.5) | 0.969 |
|  | Visit 3 | 187.4 (123.7) | 169.0 (112.5) | 0.499 |
|  | Visit 4 | 220.1 (162.8) | 195.2 (192.1) | 0.543 |
|  | Visit 5 | 182.8 (112.2) | 167.3 (121.7) | 0.564 |
| Basophils (/μL) | Visit 2 | 48.4 (23.5) | 48.4 (22.3) | 1.000 |
|  | Visit 3 | 51.2 (49.5) | 47.3 (21.7) | 0.659 |
|  | Visit 4 | 47.3 (20.4) | 47.8 (22.6) | 0.920 |
|  | Visit 5 | 46.6 (20.7) | 44.2 (19.9) | 0.612 |
| ALP (U/L) | Visit 2 | 75.8 (20.6) | 77.6 (22.8) | 0.717 |
|  | Visit 3 | 74.0 (19.0) | 77.8 (21.8) | 0.418 |
|  | Visit 4 | 78.9 (21.7) | 77.9 (22.1) | 0.843 |
|  | Visit 5 | 78.2 (22.3) | 78.0 (21.5) | 0.971 |
| LD (U/L) | Visit 2 | 201.7 (29.2) | 204.1 (26.7) | 0.719 |
|  | Visit 3 | 214.1 (36.6) | 212.2 (29.9) | 0.808 |
|  | Visit 4 | 205.6 (28.6) | 212.1 (27.6) | 0.321 |
|  | Visit 5 | 202.7 (27.0) | 208.2 (28.5) | 0.386 |
| LAP (U/L) | Visit 2 | 56.7 (9.9) | 58.9 (10.6) | 0.346 |
|  | Visit 3 | 57.2 (13.3) | 60.1 (10.8) | 0.297 |
|  | Visit 4 | 58.6 (11.6) | 59.7 (10.2) | 0.676 |
|  | Visit 5 | 57.2 (12.2) | 58.7 (9.6) | 0.560 |
| Total bilirubin (mg/dL) | Visit 2 | 0.8 (0.2) | 0.9 (0.4) | 0.154 |
|  | Visit 3 | 0.8 (0.3) | 0.9 (0.3) | 0.208 |
|  | Visit 4 | 0.8 (0.4) | 0.9 (0.3) | 0.113 |
|  | Visit 5 | 0.8 (0.3) | 0.9 (0.4) | 0.484 |
| Data are expressed as mean (SD). The differences between the groups were determined using Welch’s t-test.  Abbreviations: ALP, alkaline phosphatase; LD, lactate dehydrogenase; LAP, leucine aminopeptidase | | | | |
