# Supplementary material for: Effects of broccoli sprout supplements enriched in glucoraphanin on liver functions in healthy middle-aged adults with high-normal serum hepatic biomarkers: A randomized controlled trial
Source: Front Nutr. 2022 Dec 22;9:1077271. doi: 10.3389/fnut.2022.1077271 (PMC9813215; doi:10.3389/fnut.2022.1077271)
Supplement: Supplementary file 2 [file Presentation_1.PPTX]

## Slide 1
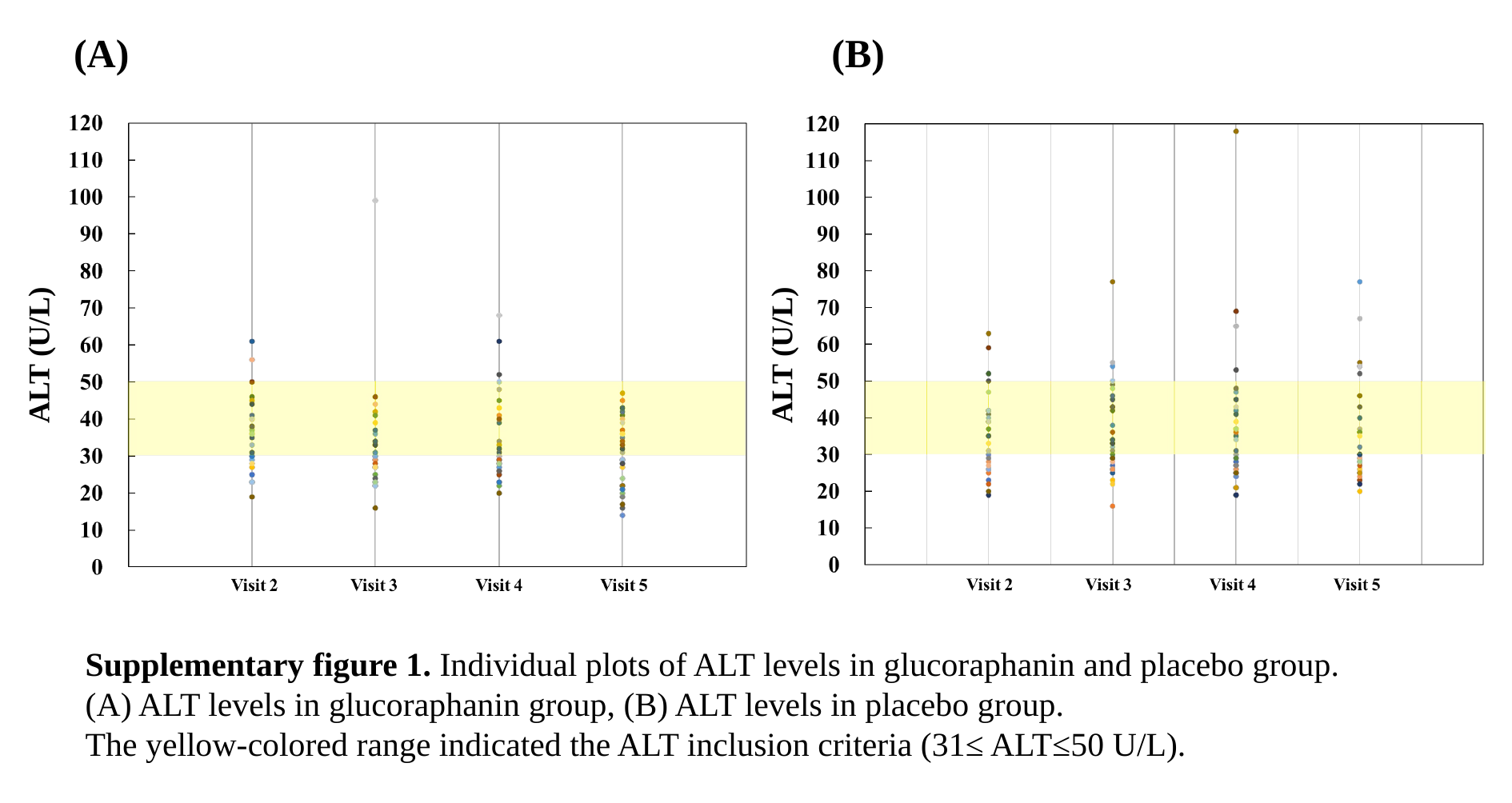

(A)
(B)
ALT (U/L)
ALT (U/L)
Supplementary figure 1. Individual plots of ALT levels in glucoraphanin and placebo group.
(A) ALT levels in glucoraphanin group, (B) ALT levels in placebo group.
The yellow-colored range indicated the ALT inclusion criteria (31≤ ALT≤50 U/L).
